# ChatGPT for Patients: A Comprehensive Study on Atrial Fibrillation Awareness

**DOI:** 10.19102/icrm.2024.15072

**Published:** 2024-07-15

**Authors:** Rahul Vyas, Arpita Pawa, Chanza Shaikh, Anaiya Singh, Hetvi Shah, Shubhika Jain, Vijaywant Brar

**Affiliations:** 1 Department of Internal Medicine, LSU, Shreveport, LA, USA; 2 Department of Internal Medicine, Willis-Knighton Health System, Shreveport, LA, USA; 3 R.C.S.M Government Medical College, Kolhapur, Maharashtra, India; 4 Kasturba Medical College, Manipal, Karnataka, India; 5 Department of Cardiology, LSU, Shreveport, LA, USA

**Keywords:** Artificial intelligence, atrial fibrillation, ChatGPT

## Abstract

Due to the intricate nature of atrial fibrillation (AF), the diagnostic process often gives rise to a spectrum of concerns and inquiries. A 20-question survey on AF, covering general concerns, diagnosis, treatment, and post-diagnosis inquiries, was conducted via Google Forms (Google LLC, Mountain View, CA, USA). The questions were input into the Chat Generative Pre-trained Transformer (ChatGPT) system (OpenAI LP, San Francisco, CA, USA) in November 2023, and the responses were meticulously collated within the same Google Forms. The survey, involving 30 experienced physicians, including 22 cardiologists and 8 hospitalists, practicing for an average of 18 years, assessed artificial intelligence (AI)-generated responses to 20 medical queries. Out of 600 evaluations, “excellent” responses were most common (29.50%), followed by “very good” (26%), “good” (19.50%), and “fair” (17.3%). The least common response was “poor” (7.67%). Questions were categorized into “general concerns,” “diagnosis-related,” “treatment-related,” and “post-diagnosis general questions.” Across all categories, >50% of experts rated responses as “excellent” or “very good,” indicating the potential for improvement in the AI’s clinical response methodology. This study highlights the efficacy of ChatGPT as an AF informational resource, with expert-rated responses comparable to those of clinicians. While proficient, concerns include infrequent updates and ethical considerations. Nevertheless, it underscores the growing role of AI in health care information access.

## Introduction

Atrial fibrillation (AF) is the most common cardiac arrhythmia characterized by irregular electrical activity in the atria, leading to irregular and fast ventricular rates.^1^ Over the past few decades, the incidence and prevalence of AF have increased substantially, with estimates suggesting a threefold increase in global prevalence over the last 50 years.^2^ The economic burden of AF in the United States is estimated to be $6–$26 billion annually.^3^ Factors such as advancing age, underlying structural heart disease, hypertension, diabetes mellitus, and smoking are some of the most common risk factors associated with the development of AF. of note, patients commonly present with chest discomfort, palpitations, fatigue, and dizziness/syncope, causing substantial distress.^4^ Although the electrocardiogram has historically been the gold standard for AF diagnosis, contemporary non-invasive heart monitoring devices are progressively being used for early detection and diagnosis.^1,2^ Most of these patients require lifelong treatment for rate/rhythm control and anticoagulants.^1^ Given the complex nature of the disease, non-specific presenting symptoms, and the need for long-term treatment, the diagnosis is often accompanied by various apprehensions and questions. In the era of easily accessible information, tools like Chat Generative Pre-trained Transformer (ChatGPT) (OpenAI LP, San Francisco, CA, USA) hold the potential to address concerns and serve as a bridge between health care providers and patients.^5^ Through this study, we aimed to evaluate the accuracy of responses provided by the ChatGPT interface to common questions about the diagnosis and treatment of AF. To assess the integrity of the responses generated by ChatGPT, we engaged the expertise of attending physicians, who diligently analyzed the responses and contributed to the conclusions. This study intends to highlight the potential role of artificial intelligence (AI) in health care.

## Methods

A comprehensive 20-question survey addressing the fundamental aspects of AF was formulated and administered through Google Forms. The questions were formulated based on frequently asked questions available on websites such as the American Heart Association (AHA), ensuring comprehensive coverage of the fundamental aspects related to AF.^6^

The survey, totaling 20 questions, was distributed to a pool of 70 physicians, 30 of whom responded. Out of the 30 doctors who responded, 22 were cardiologists and 8 were hospitalists. The participating physicians were selected by a simple random sampling method.

The survey encompassed inquiries categorized into four distinct sections—namely, general concerns regarding AF, the diagnostic process for AF, available treatment modalities for AF, and inquiries that commonly arise post-diagnosis. These questions were input into the ChatGPT system in November 2023, with the subsequent responses meticulously collated within the same Google Forms. To ascertain the reliability and credibility of the information provided by ChatGPT, a panel of 30 physicians who voluntarily participated in the survey assessed the responses. Using a Likert scale ranging from “poor” to “excellent,” the doctors evaluated the quality of information provided by ChatGPT across the spectrum of AF-related queries. The resultant data facilitated the establishment of a consensus among the physicians concerning the efficacy of ChatGPT as a source of information for patients with AF. Additionally, during subsequent discussions, any identified shortcomings or limitations in the utility of ChatGPT in addressing the specific informational needs of patients on AF were thoroughly examined. This approach not only helped assess the precision of the information provided by ChatGPT but also offered insight into the possible advantages and limitations linked to relying on AI as a dependable source of information on AF for patients.

No informed consent or ethical approvals were required for this study.

## Results

The survey aimed to assess the quality of AI-generated responses to AF-related questions as perceived by 30 experienced physicians. The participants, having practiced for an average of 18 (±10.2) years with a mean age of 49 (±11.1) years, provided 600 evaluations of computerized responses to 20 medical queries **(Table 1)**. A higher proportion (n = 20, 66.7%) of them affirmed ChatGPT as a reliable information source for patients seeking accuracy, while opinions on integrating ChatGPT into electronic patient portals for effective patient query handling were evenly split (yes, n = 15, 50%; no, n = 15, 50%). Additionally, 60% of participating physicians concurred that ChatGPT’s responses to patient queries were comparable to those from practicing clinicians, whereas 20% (n = 6) disagreed or somewhat agreed. Notably, a majority (yes, n = 12, 40%; they will coexist, n = 11, 36.7%; no, n = 7, 23.3%) foresaw that AI platforms like ChatGPT may eventually surpass conventional search engines such as Google as the preferred conduit for online patient information **(Table 2)**.

Out of the 600 total quality responses recorded, the most common response was “excellent” (n = 177, 29.50%), followed by “very good” (n = 156, 26%) and then “good” (n = 117, 19.50%) and “fair” (n = 104, 17.3%). The question “What treatment options do I have after being diagnosed with atrial fibrillation?” received the most responses in the “excellent” category, and the response generated by ChatGPT was as follows: “The treatment of Afib [atrial fibrillation] aims to control the heart rate, restore normal rhythm, and prevent complications such as stroke. Here are the main treatment options for Afib:

- ***Medications: anti-arrhythmic drugs:*** These medications help regulate the heart’s rhythm. Examples include amiodarone, flecainide, propafenone, and sotalol.
- ***Rate-control drugs:*** These medications slow down the heart rate. Common options include β-blockers (e.g., metoprolol, carvedilol) and calcium channel blockers (e.g., diltiazem, verapamil).
- ***Anticoagulants:*** Afib increases the risk of blood clots and stroke. Anticoagulant medications such as warfarin, dabigatran, rivaroxaban, and apixaban are often prescribed to reduce this risk.
- ***Cardioversion:*** This procedure involves restoring the heart’s normal rhythm using electrical shocks or medications. It can be done in emergency situations or as a planned procedure.
- ***Ablation: catheter ablation:*** In this procedure, a catheter is used to deliver radiofrequency energy or cryotherapy to destroy or isolate areas in the heart causing abnormal electrical signals.
- ***Maze procedure:*** This is a surgical ablation technique where a series of incisions are made in the atria to create a maze-like pattern. This disrupts abnormal electrical pathways, restoring a normal heart rhythm. The maze procedure is often done during other heart surgeries.
- ***Implantable devices: pacemaker:*** If Afib is associated with a slow heart rate, a pacemaker may be implanted to regulate and maintain a normal heart rate.
- ***Implantable cardioverter-defibrillator (ICD):*** For those at risk of life-threatening arrhythmias, an ICD may be recommended.
- ***Lifestyle changes:*** Adopting a heart-healthy lifestyle can be crucial. This includes regular exercise, a balanced diet low in salt and saturated fats, maintaining a healthy weight, limiting alcohol intake, and avoiding smoking.
- ***Blood pressure management:*** Controlling blood pressure is essential, as hypertension is a common contributing factor to Afib.

It’s important to note that the choice of treatment depends on factors such as the severity of symptoms, the underlying cause of Afib, and individual patient characteristics. The treatment plan is typically personalized, and patients should work closely with their health care team to determine the most suitable approach for their specific situation. Regular follow-ups are crucial to monitor the effectiveness of the chosen treatment and make any necessary adjustments.”

**Figure 1:**
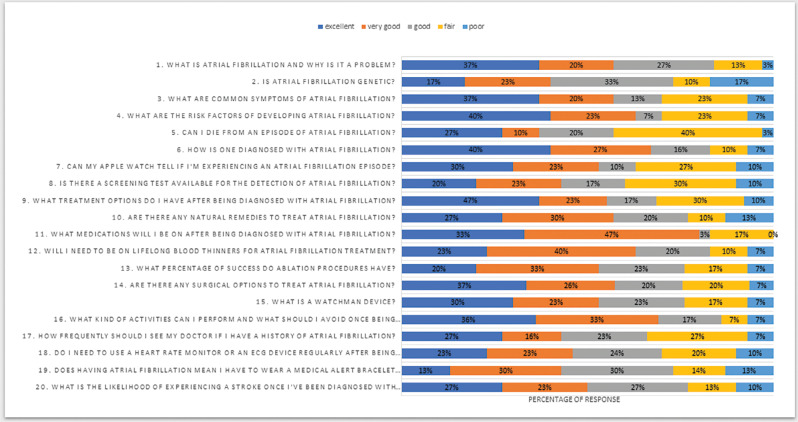
Clustered bar graph showing the percentages of quality grades assigned to each question.

The least common response was “poor” (n = 46, 7.67%) **(Figure 1)**, and the question “Is atrial fibrillation genetic?” had the highest number of responses in the “poor” category. The response generated by ChatGPT to this question was as follows: “Atrial fibrillation (Afib) can have a genetic component, but its development is influenced by a combination of genetic and environmental factors. However, the exact genetic factors contributing to Afib are complex and not fully understood.”

Furthermore, the questions were divided into four main categories. In the chronological sequence, questions 1–5 were categorized into “general concerns”; questions 6–10 depicted “diagnosis-related questions”; questions 11–15 were classified into “treatment-related queries”; and questions 16–20 related to the “once diagnosed, general questions by patients” category. In all four categories, ≥50% of experts rated the responses as “excellent” or “very good,” while the “poor” rating was noted in 19 out of the 20 questions **(Figure 1)**. The results thus demonstrate there is substantial room for enhancing AI’s clinical response methodology in the future.

## Discussion

This distinctive study highlights the role of AI tools like ChatGPT as an informational source for patients with AF. Using technology as an ally, patients can be encouraged to use ChatGPT to educate themselves about AF, albeit cautiously. In our study, most of the feedback received from experts rated the responses generated by ChatGPT as excellent or very good. Most of them believed that the quality of the responses was comparable to that of the responses of actual clinicians. They believed the answers were technically accurate yet worded in an easy-to-understand manner. The ability of ChatGPT to produce high-quality responses is attributed to its use of unsupervised pre-training methods, which allow it to access a vast amount of text-based data and formulate responses conversationally. We found that the answers provided by ChatGPT were comprehensive while also routinely recommending close monitoring by health care professionals. Especially when first breaking down the diagnosis of a new disorder like AF, interfaces like ChatGPT can efficiently help bridge the gap between providers and patients by summarizing key points effectively for more transparent communication, reducing patient apprehension.

However, these tools are not without their share of concerns; most of the answers provided by ChatGPT are not constantly updated according to the most recent advances and guidelines and are not based on regional practices. Specific questions produce different answers when reframed; hence, the reproducibility of information remains challenging. The ethical aspect of using AI remains a gray area with no consensus within health care.^5,7^ To sum up, the present research simply emphasizes the general usefulness of ChatGPT as a significant source of information for patients recently diagnosed with AF. This reflects the increasing incorporation of AI systems in health care, which could revolutionize how patients obtain online information.

### Limitations

Out of the 70 physicians approached for this study, only 30 responded. This sample size may not fully encompass the perspectives and opinions of the health care community at large, and some degree of variability may be expected if a larger physician population is interviewed.

Participating physicians provided a subjective evaluation of responses, which may vary with personal bias or preferences. They may also be more inclined to rate AI-generated responses positively due to the novelty of newer technology, thereby overestimating the efficacy of AI.

## Conclusion

In conclusion, this study emphasizes the potential of AI tools, exemplified by ChatGPT, in providing valuable information to patients with AF. The majority of surveyed physicians acknowledged the accuracy and usefulness of ChatGPT’s responses, recognizing its role in enhancing patient education and communication. However, limitations, such as the need for continuous updates, regional variability, and ethical considerations, should be addressed. As AI integration in health care advances, using such tools cautiously can contribute to more informed and engaged patient populations.

## Figures and Tables

**Table 1:** Quality of ChatGPT Responses to Questions Pertaining to AF

Characteristic	n = 30 (%)
Age of cardiologists (years)	
Mean (SD)	49 (11.1)
Median (IQR)	50 (32–68)
Years in practice	
Mean (SD)	18 (10.2)
Median (IQR)	20 (4–36)
Practice setting	
Academic center, n (%)	14 (46.7)
Non-academic center, n (%)	16 (53.3)

*Abbreviations:* AF, atrial fibrillation; IQR, interquartile range; SD, standard deviation.

**Table 2:** Overall Feedback by Experts on the Performance of ChatGPT Relative to Information About AF

Opinion	n (%)
ChatGPT serves as a reliable source of information catering to patients seeking accurate information.	
Yes	20 (66.7)
No	10 (33.3)
Integrating ChatGPT into electronic patient portals will emerge as a favorable initial approach for addressing patient queries effectively.	
Yes	15 (50.0)
No	15 (50.0)
ChatGPT’s responses to patient queries demonstrate a level of comparability to those offered by practicing clinicians.	
Yes	18 (60.0)
No	6 (20.0)
Somewhat	6 (20.0)
In the foreseeable future, AI platforms like ChatGPT may replace conventional search engines such as Google in becoming the favored conduit for online patient information.	
Yes	12 (40.0)
No	7 (23.3)
They will coexist	11 (36.7)

*Abbreviations:* AF, atrial fibrillation; ChatGPT, Chat Generative Pre-trained Transformer.
